# Intrinsic Structural Disorder Confers Cellular Viability on Oncogenic Fusion Proteins

**DOI:** 10.1371/journal.pcbi.1000552

**Published:** 2009-10-30

**Authors:** Hedi Hegyi, László Buday, Peter Tompa

**Affiliations:** 1 Institute of Enzymology, Biological Research Center, Hungarian Academy of Sciences, Budapest, Hungary; 2 Department of Medical Chemistry, Semmelweis University Medical School, Budapest, Hungary; Fox Chase Cancer Center, United States of America

## Abstract

Chromosomal translocations, which often generate chimeric proteins by fusing segments of two distinct genes, represent the single major genetic aberration leading to cancer. We suggest that the unifying theme of these events is a high level of intrinsic structural disorder, enabling fusion proteins to evade cellular surveillance mechanisms that eliminate misfolded proteins. Predictions in 406 translocation-related human proteins show that they are significantly enriched in disorder (43.3% vs. 20.7% in all human proteins), they have fewer Pfam domains, and their translocation breakpoints tend to avoid domain splitting. The vicinity of the breakpoint is significantly more disordered than the rest of these already highly disordered fusion proteins. In the unlikely event of domain splitting in fusion it usually spares much of the domain or splits at locations where the newly exposed hydrophobic surface area approximates that of an intact domain. The mechanisms of action of fusion proteins suggest that in most cases their structural disorder is also essential to the acquired oncogenic function, enabling the long-range structural communication of remote binding and/or catalytic elements. In this respect, there are three major mechanisms that contribute to generating an oncogenic signal: (i) a phosphorylation site and a tyrosine-kinase domain are fused, and structural disorder of the intervening region enables intramolecular phosphorylation (e.g., BCR-ABL); (ii) a dimerisation domain fuses with a tyrosine kinase domain and disorder enables the two subunits within the homodimer to engage in permanent intermolecular phosphorylations (e.g., TFG-ALK); (iii) the fusion of a DNA-binding element to a transactivator domain results in an aberrant transcription factor that causes severe misregulation of transcription (e.g. EWS-ATF). Our findings also suggest novel strategies of intervention against the ensuing neoplastic transformations.

## Author Summary

Chromosomal translocations generate chimeric proteins by fusing segments of two distinct genes and are frequently associated with cancer. The proteins involved are large and fairly heterogeneous in sequence and typically have only a few dispersed structural domains connected by long uncharacterized regions. It has never been studied from a structural perspective how these chimeras survive losing significant portions of the original proteins and acquire new oncogenic functions. By analyzing a collection of 406 human translocation proteins we show here that the answer to both questions lies to a large extent in the high level of structural disorder in the fusion partner proteins (on average, they are twice as disordered as all human proteins). The translocation breakpoints usually avoid globular domains. In rare cases when a globular domain is truncated by the fusion, it happens at a location in the domain where the hydrophobicity exposed by the split is favorable (i.e., not too high). Disorder on average is significantly higher in the vicinity of the breakpoint than in the rest of the fusion proteins. Disorder also plays a pivotal role in the acquired oncogenic function by bringing distant/disparate fusion segments together that enables novel intra- and/or intermolecular interactions.

## Introduction

Chromosomal translocations are the major genetic aberration in cancers, such as leukemias, lymphomas and sarcomas [1]–[4]. Translocation links two distinct chromosomes, and either fuses one gene to the regulatory region of another gene, or results in a chimera by the fusion of two unrelated genes. The resulting misregulation of the expression of a normal gene or appearance of a unique fusion protein is the cause of neoplastic transformations in many cases. Molecular understanding of the translocation event is of paramount importance in devising strategies against these diseases [3],[4]. Translocation has been extensively studied at the genetic level, leading to the recognition that its primary cause is a double-strand break (DSB) of DNA, erroneously repaired by joining two remote chromosomal segments [1]. Fusion events have also been well characterized in terms of the functions of genes/gene products involved. A dominance of DNA-binding and transcription regulatory functions have been observed, whereas at the domain level kinases and DNA-binding motifs occur most frequently [2], [5]–[7].

Much less is known about the structural implications of protein fusion. The proteins involved are often quite long and complex, heterogeneous in sequence and structure, and contain only a few dispersed domains, usually avoided by the translocation breakpoints [3],[4],[8]. This is particularly true of proteins that appear in chromosomal translocations recurrently, such as MLL [9], CBP [8], or EWS [10]. This has led to the suggestion that the cellular survival of the protein chimera can be explained by its structural disorder, because it enables the cellular viability of a protein generated from segments of two unrelated proteins [8]. The rationale of this notion rests on the prevalence of intrinsically disordered/unstructured proteins (IDPs/IUPs) or protein regions (IDRs), which exist and function without well-defined 3D structures [8],[11],[12]. Structural disorder reaches high levels in proteins of regulatory and transcriptional functions [13],[14], and shows significant evolutionary increase in eukaryotes, compared to prokaryotes [15]. IDPs/IDRs can function as disordered linkers, but most often they carry out their functions by molecular recognition, in which they bind other protein(s), DNA or RNA in a binding-induced folding process [8],[11]. Disorder plays a prominent role in cancer-associated proteins [13] and alternative splicing (AS), a process which generates distinct protein products from the same initial transcript [16]. AS may connect disjoint segments of proteins into a viable protein product, in a process conceptually similar to protein fusion. In fact, for two fusion products, EFP [10] and CBP-NOZ [8], the involvement of structural disorder in fusion has been explicitly stated. Nevertheless, its role in either the cellular survival of the fusion product or the ensuing oncogenic function has neither experimentally nor statistically been addressed in these or other works.

Motivated by these inferences, we have tested the association of structural disorder with chromosomal translocations. We collected 406 human fusion proteins (255 with identifiable breakpoints), and analyzed their disorder by the IUPred prediction algorithm [17]. We found that fusion proteins have a very high level of disorder, their translocation breakpoints tend to avoid domains, and disorder appears to play a major role in their oncogenic functions. These findings shed new light on the structural background of how protein products generated by chromosomal translocations are selected for by cellular proliferation and clonal expansion in cancer, and suggest novel strategies of intervention against the ensuing oncogenic transformations.

## Results

### Length dependence of translocation

**Figure 1:**
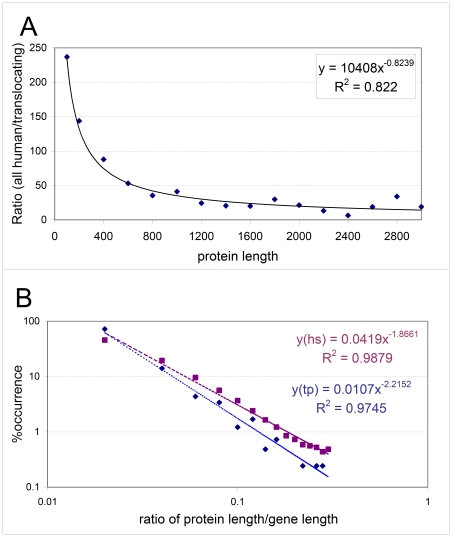
Length of proteins and genes involved in translocation. **(A)** Ratio of all human and translocating proteins as a function of protein length, shown by increments of 100 amino acids. The data were fitted with a power function. **(B)** The percentage distribution of the ratio of protein and gene length for translocating partner proteins (cyan) and all human proteins (magenta). Both functions show a linear tendency when represented on a logarithmic scale (as shown here), which is characteristic of the power law function. The explicit trendline is also shown for both sets of proteins.

As suggested in the Introduction, translocation is initiated by the DSB of DNA, which may occur either at random or in conjunction with some special feature of DNA sequence/structure. Both scenarios suggest that longer proteins/genes are more likely to undergo DSB and subsequent translocation. This has already been shown in a study on a smaller database of translocation proteins among 291 cancer proteins [18], which suggested that translocated cancer proteins tend to have longer genes, whereas cancer genes with point mutations tend to encode longer proteins, both significantly longer than average human genes/proteins. Because of the noted increase of structural disorder with the length of proteins [19],[20], we repeated this analysis on our database of 406 proteins involved in translocations. To this end, the frequency of translocation as a function of the length of the protein (Figure 1A) or the length of the gene (Figure S1) in question was determined. Both functions have an inverse relationship, which suggests that proteins/genes become involved in translocation event(s) roughly in proportion to their length. Nevertheless, the frequency of translocation increases with protein length at a greater power than with gene length (0.82 vs. 0.59), which suggests that additional structural/functional forces of selection besides mere chance of random DNA DSB events operate at the protein level. Figure 1B shows the distribution of the ratio of protein and gene length for both proteins involved in translocation and all human proteins. Again, a clear difference between the two sets of proteins, where the translocating protein set falls off with a steeper slope, indicate an even greater relative gene length for this set of proteins, which suggests that additional selection forces act at the protein level.

### Disorder in translocation proteins

**Figure 2:**
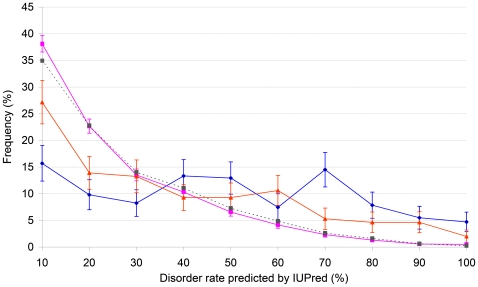
Structural disorder in proteins involved in translocation. Intrinsic disorder percentage distribution of translocating proteins. The percent intrinsic disorder of proteins involved in chromosomal translocation with (255, blue diamonds) and without (151, orange triangles) a known breakpoint and all human proteins in Swissprot (18,609, magenta squares), is shown. Bin size is 10%. Each of the 406 translocating proteins represents a different gene, the longest known protein isoform was chosen for each. Gray dotted line shows the disorder distribution of 500 randomly selected sets of 255 human Swissprot proteins with length that match that of the 255 translocating proteins with breakpoints.

Human translocation proteins have an extensive disorder predicted by IUPred [17], with a significant excess of proteins of 70–80% disorder (Figure 2). Comparing the distributions with a chi-square test we found that proteins with an established translocation breakpoint differ very significantly from that of all Swissprot human proteins (p-value<1e-14) whereas those without a known breakpoint differ from all of Swissprot less significantly (p<0.00058). The two sets of translocating proteins also differ from each other (p<0.00667). The mean disorder of all human proteins is 20.7% whereas that of proteins with known translocation breakpoint(s) is 43.3%. Translocation proteins without a known breakpoint have a small local maximum at 60% disorder probably reflecting so far undiscovered breakpoint(s) in some of them, whereas for others the breakpoint is located outside the coding region. The mean disorder for this latter set is 32.1%. The disorder distribution of 500×255 randomly drawn human Swissprot proteins matched in length to translocation proteins with breakpoint is very similar to the total of 18,609 human proteins currently in Swissprot (with a mean disorder 21.9%). The slight difference 1.2% could be attributed to longer proteins having somewhat higher disorder also observed by others [19],[20]. This bias, however, does not account for the elevated level of disorder in translocating proteins (cf. Figure 2).

The ratio of the number of all human proteins divided by all translocating proteins with a breakpoint as a function of disorder is shown in Figure S2. The figure shows the increasing frequency of protein translocation with increasing disorder (to be more precise, the chance of such proteins surviving the translocation event increases). As the best fitting trend line is a parable (with an almost perfect fit, R^2^ = 0.9587) the relationship appears quadrant, i.e. twice as much disorder entails four times more frequent translocation (with the exception of the disorder range 90–100% where the tendency seems to reverse itself). All the proteins in this set, their disorder, length and breakpoints are listed in Table S1.

### Disorder at the breakpoint

**Figure 3:**
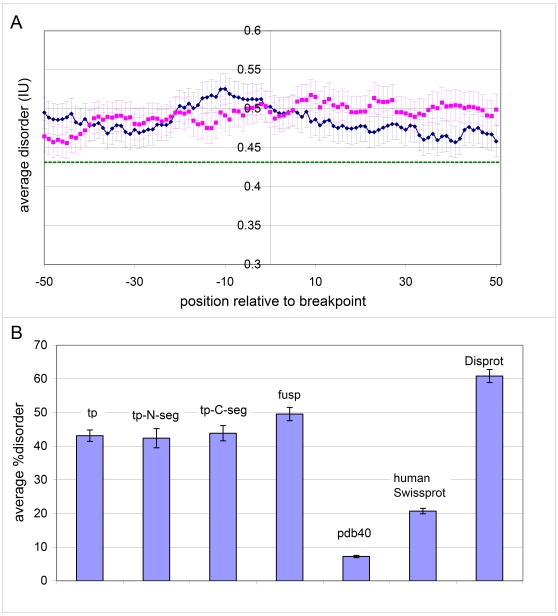
Structural disorder in translocation proteins and other proteins. **(A)** Structural disorder in translocation proteins was predicted separately for the protein providing the N-terminal (blue) and C-terminal (magenta) segment of the fusion protein generated by the translocation event. Error bars represent SDs. **(B)** Mean disorder values for translocating proteins (**tp**), N-, and C-terminal segments (**tp-N-seg**, **tp-C-seg**) and fusion products (**fusp**) were predicted with IUPred. For reference, the mean disorder of proteins in the PDB database (**pdb40**, 40% non-redundant in sequence similarity), all human proteins in Swissprot (**SwissProt**) and experimentally determined disordered proteins/segments in DisProt (**DisProt**), are also shown. Error bars represent SDs.

To address if disorder at the point of fusion is preferred for the survival of the protein chimera we assessed the average level of disorder in translocation proteins. With respect to the actual breakpoint and its vicinity, we predicted disorder around the breakpoint separately for the N- and C-terminal partners within the range of [−50,50] (Figure 3A). In the N-terminal partners the highest values appear in the [−20, 0] region, whereas the values to the right of the breakpoint gradually fall off, in accord with the fact that these parts are eliminated during fusion. In the C-terminal partners it is the values right to the breakpoint that increase in the parts retained in the fusion products. Averaging the averages in the [−50,50] range around the breakpoint we found that the total average for this region is 0.49 (with SD = 0.016), which is significantly higher than in the rest of these proteins (0.43, cf. Figure 2 and Figure 3B; the difference is more than 3 SD-s).

The mean values for the overall disorder of the translocation partners are shown in Figure 3B (with SDs indicated). Apparently they have a very high level of disorder, e.g. more than twice as high as for all human proteins in Swissprot, with a tendency of fusion products to have an even higher one. Comparing them to proteins experimentally determined to be fully disordered (e.g. those in DisProt) and fully ordered (e.g. those in PDB), it can be ascertained that selection following chromosomal translocation strongly favors fusion proteins in which structural disorder dominates.

### Translocation and domains in the fusion partner proteins

A clear implication of the above findings is that a protein product is highly disfavored if its site of joining falls in ordered domains, which would most probably lead to the creation of structurally aberrant chimeras. To check this assumption, we have analyzed translocation proteins for the occurrence of Pfam domains. We found that the average coverage of translocation proteins by Pfam domains is 36.3%, whereas this value for a human Swissprot protein is 42.5%. On the other hand, in proteins generated by fusion (where each gene pair is considered only once, with the longest fusion protein for each) Pfam coverage decreases to 30.9%. A chi-square test showed that all three values (and the underlining distributions) differ significantly, each set differs from the total of human Swissprot with p-value<1e-9, and even the translocation partner proteins and the fusion proteins differ from one another regarding their coverage by Pfam domains with p-value = 0.012.

To check if the breakpoint tends to “avoid” Pfam domains (i.e. such proteins are selected against by cellular proliferation and clonal expansion), we compared the number of actually truncated domains with that of a set of proteins with a randomly generated breakpoint. We found that while the actual number of truncated domains in the 255 translocation partner proteins with an established breakpoint was 48, the random breakpoints (repeating the random selection process 200 times) resulted in 76.2 domain truncations on average with a standard deviation of 5.6, that is, the difference is highly significant, with a Z-value of 4.87.

In addition, the average disorder of Pfam domains occurring in the fusion products is 14.3% (not significantly different from that in all human proteins, 13.1%), whereas the average disorder of Pfam domains actually truncated is significantly higher, 26.8%. In cases where the missing or retained fragment is more than 10% of the original size of the domain, this disorder value increases to 29.6%.

### Fate of truncated domains in the fusion products

A closer look at actually truncated Pfam domains located in the fusion proteins (Table 1) provides further evidence for the structural bias of protein products generated by fusion events (for a compendium of all Pfam domain matches where the breakpoint falls in a domain, see Table S2). In several cases the Pfam domain is actually a coiled-coil, which is structurally rather indifferent to the location of truncation. In addition, the remainder of the domain very often has a significant level of disorder, or it is apposed with a rather disordered segment on the fusion partner.

**Figure 4:**
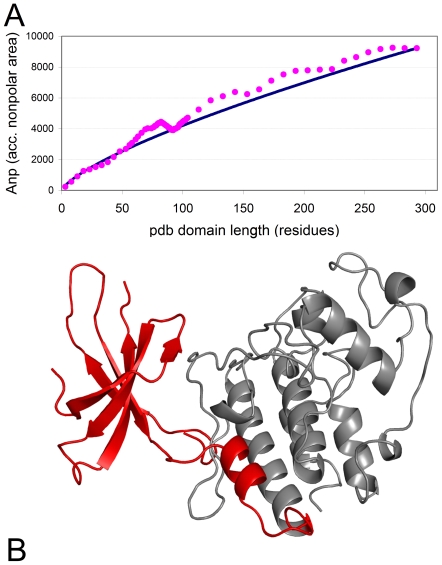
Effect of truncation on the accessible hydrophobic surface of a kinase domain. **(A)** Theoretical and actual values for the accessible nonpolar surface area (**Anp**) of a cyclin-dependent kinase. The C-terminus of the protein structure was gradually truncated and the actual values of **Anp** (full magenta circles) for the truncated fragments were determined with the CHASA program [22]. They coincide with the theoretical values for an intact domain of the same size (blue contiguous line) around residue 90. **(B)** Structure of the cyclin-dependent kinase (PDB code 1g3n, chain A). The C-terminal portion missing due to the translocation is colored grey.

**Table 1:** Truncated domains in the fusion proteins.

fusion protein	fplen	bp	Pfid	Dlen	Dbeg	Dend	N/C	Dfract	IUleft	IUright	Pfam Desc	Mode of Survival?
**MLL-ELL**	1966	**1406**	PF10390	237	4	237	**C**	0.99	**80.1**	*43.5*	RNA pol II ef	no PDB
**Col1A1-PDGFB**	488	**269**	PF04692	76	2	76	**C**	0.99	**97.3**	*64.3*	Platelet gf	almost full domain
**CBFB-MYH11**	553	**165**	PF02312	171	1	165	**N**	0.96	*38.2*	**61.4**	Core bind f α	almost full domain
**RUNX1-CBFA2T3**	806	**204**	PF00853	135	1	130	**N**	0.96	*41.9*	**67.4**	Runt domain	almost full domain
**NSD1-ANKRD28**	811	**412**	PF00023	33	3	33	**C**	0.94	**38.6**	*24.0*	Ankyrin	short repeats
**TPM4-ALK**	784	**221**	PF00261	237	1	210	**N**	0.89	*52.1*	**24.4**	Tropomyosin	coiled-coil
**DAZAP1-MEF2D**	390	**154**	PF00076	70	1	60	**N**	0.86	*50.0*	**63.5**	RNA rec motif	2dgs_A termini IUP (11, 14 aa)
**IL2-TNFRSF17**	298	**117**	PF00715	144	1	123	**N**	0.85	*14.8*	**12.5**	Interleukin 2	artefact
**MN1-ETV6**	1653	**1256**	PF02198	87	14	87	**C**	0.85	**50.7**	*30.9*	SAM domain	1×66_A N-terminal 18 aa IUP
**TMPRSS2-ETV1**	422	**5**	PF04621	333	61	333	**C**	0.82	**62.3**	*55.1*	PEA3 ETS tf N	no PDB
**DDX10-NUP98**	1462	**219**	PF00270	171	1	133	**N**	0.78	*22.7*	**61.7**	DEAD b h-ase	***1vec_A 151***
**CDK6-MLL**	2599	**123**	PF00628	53	13	53	**C**	0.77	**29.0**	*12.5*	PHD-finger	***1weu_A 12, 17***
**E2A-PRL**	825	**483**	PF03792	200	56	200	**C**	0.73	**65.7**	*48.4*	PBC domain	no PDB
**CLTC-TFE3**	1212	**932**	PF00637	140	1	101	**N**	0.72	*19.7*	**42.3**	Clathrin rep	elongated coil of a-helices
**MLL-AFF1**	2308	**1444**	PF05110	1201	338	1201	**C**	0.72	**67.2**	*72.1*	AF-4 oncoprot	no PDB
**RUNX1-AFF3**	1184	**322**	PF05110	1205	341	1205	**C**	0.72	**54.3**	*62.9*	AF-4 oncoprot	no PDB
**MYH9-ALK**	2207	**1644**	PF01576	858	1	605	**N**	0.71	*55.6*	**41.4**	Myosin tail	no PDB
**RABEP1-PDGFRB**	1317	**738**	PF09311	196	1	127	**N**	0.65	*24.8*	**15.4**	Rab5 binding	coiled-coil
**NPM-RAR**	563	**133**	PF03066	199	1	121	**N**	0.61	*39.6*	**21.2**	Nucleoplasmin	nucleophosmin 2p1b_H, 122 aa
**COL6A3-CSF1**	2089	**1738**	PF00092	174	1	100	**N**	0.57	*40.7*	**77.8**	VWA	***1dzi_A 104,114***
**Col1A1-PDGFB**	488	**269**	PF01391	60	1	34	**N**	0.57	*97.3*	**64.3**	Collagen	coiled-coil, repeat
**ETV6-JAK2**	622	**154**	PF07714	261	**118**	**261**	**C**	0.55	**31.8**	*28.0*	Tyr kinase	***2f4j_A 157, 167***
**MLL-FOXO3**	1910	**1444**	PF00250	98	50	98	**C**	0.51	**49.1**	*58.0*	Fork head	***1jxs_A 43, 53, 63***
**MSN-ALK**	1005	**448**	PF00769	240	1	111	**N**	0.46	*62.3*	**34.9**	Ezrin	***1e5w_A 106, 206, 286, 306***
**CBFB-MYH11**	553	**165**	PF01576	858	512	858	**C**	0.40	**38.2**	*61.4*	Myosin tail	no PDB
**CDK6-MLL**	2599	**123**	PF00069	288	1	114	**N**	0.40	*29.0*	**12.5**	Protein kinase	***1g3n_A 89, 94, 104***
**COL6A3-CSF1**	1571	**1211**	PF05337	268	181	268	**C**	0.33	**32.6**	*81.2*	Mphag CSF1	no PDB
**ATIC-ALK**	792	**229**	PF01808	328	1	96	**N**	0.29	*38.2*	**36.7**	AIC/IMPCHase	1p4r_A 200(scop)
**RPN1-EVI1**	1156	**87**	PF04597	429	1	59	**N**	0.14	*26.8*	**31.6**	Ribophorin I	no PDB
**MLL-CALM**	1803	**1406**	PF07651	267	237	267	**C**	0.12	**71.6**	*58.8*	ANTH domain	***1hf8_A 99, 139, 219***

Nontrivial cases of fusion proteins are shown where breakpoint falls into a Pfam domain. The abbreviated column identifiers are as follows: **Pfid**, Pfam identifier; **fplen**, fusion protein length; *bp*, breakpoint; **Dlen**, domain length, **Dbeg**, **Dend**, domain match beginning and end, respectively; **N/C**, the retained half of the truncated domain; **Dfract**, the retained fraction of the truncated domain; **IUleft**, **IUright**, the predicted disorder for the truncated domain and its “mirror” (same number of amino acids as in the truncated domain) on the opposite side of the breakpoint. In the **IUleft/IUright** columns the value for the truncated domain is *italicized* whereas the disorder value for its “mirror” is shown in **bold**. In the last column possible strategies are shown for the truncated domains to follow to avoid elimination by the proteasomal degradation system [49]. “No PDB” indicates the lack of any PDB structures associated with the protein family in question, which together with high predicted disorder values raises the suspicion of the domain being intrinsically disordered. When a PDB code is shown with a list of numbers (shown in ***bold italic***) they indicate positions in the actual domains that are presumably indifferent to truncation based on the exposed hydrophobic surface of the truncated domain (as shown in detail in Figure 4).

Furthermore, almost all cases of truncation of a globular domain (indicated in Table 1) can be structurally rationalized for the viability of the fusion product (see below), with the exception of the Interleukin 2 domain in IL2-TNFRSF17 where the fusion transcript appears not to be translated into a viable protein [21].

We analyzed in detail the severely truncated protein kinase domain in CDK6-MLL. It has low disorder and the domain retains 40% of its original size, corresponding to 114 amino acids. It appears to apply a clever strategy for cellular survival: for this domain, the accessible non-polar area resulting from sequential truncations calculated by the CHASA program [22] approaches the theoretical value of an intact domain at around 90–94 amino acids only (Figure 4A). Because this length is close to the actual 114 amino-acid truncation, this partial domain almost behaves as intact, and it is most certainly not recognized by the cellular degradation system. Figure 4B shows the missing and retained portions of the kinase fold where it also becomes clear that the fold consists of two sub-domains and only a small part of the second one is retained in the fusion protein, which probably does not fold at all. In contrast, the truncated Interleukin 2 domain does not seem to survive the loss of the last 19 amino acids of its 4-alpha-helix-like fold in the IL2-TNFRSF17 fusion transcript as the exposed hydrophobic surface of the truncated domain is prohibitively high at the site of truncation (Figure S3).

We found that several other types of domains display similar behavior, such as the von Willebrand factor A, the Fork head domain, the PHD-finger and several others (indicated in ***bold italic*** in the last column of Table 1). For the DAZAP1-MEF2D fusion protein the truncated RNA recognition motif, 2dgs_A has both its ends disordered within the last 11 and 14 amino acids. The same is true of the N-terminal 18 amino acids of the truncated 1×66_A SAM domain in MN1-ETV6 fusion protein. In the ATIC-ALK fusion protein, the truncated 1p4r_A chain (592 amino acids long) is split into two structural domains by SCOP [23] at residue 200, therefore the truncated segment is in reality only 30 amino acids long, corresponding to a fraction of 0.07 of the second SCOP domain, the latter being 392 residues long.

There are also several occurrences of consecutive short repeats and also one case of an elongated coil of alpha-helical repeats (Clathrin), which also seem to survive because repetitive structures in general are particularly well-suited for survival after truncation. Finally, there are 9 truncated domains (belonging to 7 different Pfam domain families) where there is no representative structure in the PDB, raising the suspicion (especially those with high predicted disorder values) that they could not be crystallized because they are intrinsically disordered.

### Contribution of disorder to the transforming activity of fusion proteins

**Figure 5:**
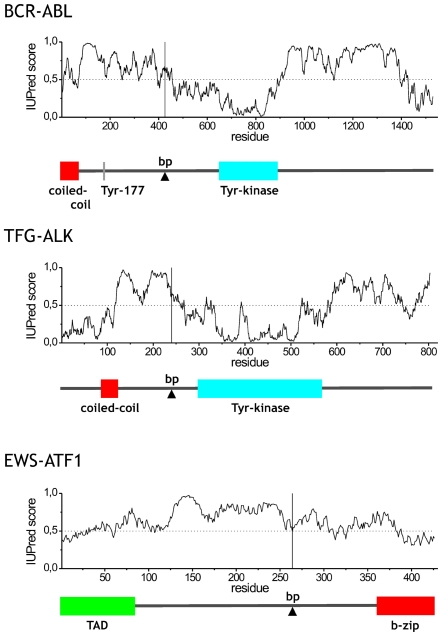
Predicted disorder and domain structure of select fusion proteins. Disorder predicted by the IUPred algorithm and domain structure identified by Pfam are shown for BCR-ABL, TFG-ALK, and EWS-ATF1, values above 0.5 are considered disordered. The position of the breakpoint is marked by a vertical line in the disorder plot whereas the elements critical for the oncogenic function are shown as colored rectangles (arrowhead for breakpoint) on the domain models below. The critical elements are connected by long segments of structural disorder.

The oncogenic function of fusion proteins suggests that disorder not only is involved in the cellular survival of the fusion product, but also in the novel function generated by the interplay of the two segments that became fused. Thus, clonal selection of cells harboring the fusion protein is promoted by the mechanism enabled by structural disorder. By considering functional information for some of the best characterized fusion proteins (Table 2) and the pattern of their predicted disorder (Figure 5), we argue that there are three basic mechanisms by which disorder contributes to the newly emerging oncogenic function.

**Table 2:** Disorder in oncogenic function of fusion proteins.

Fusion protein (breakpoint 1/breakpoint 2)	GenBank/Swissprot id (length)	Elements of oncogenic function in the fusion proteins	Distance/disorder between oncogenic elements	Reference
**BCR-ABL (426; 26)**	AAB60388 (1271)	Oligomerization domain (BCR, 1–79)	562/355	[5],[50]
	ABL1_HUMAN (1130)	Tyr kinase (ABL, 642–893)		
**NPM-ALK (117; 1057)**	NPM_HUMAN (294)	Oligomerisation domain (NPM, 1–117)	60/2	[26]
	ALK_HUMAN (1620)	Tyr kinase (ALK, 176–443)		
**EML4-ALK (496; 1057)**	EMAL4_HUMAN (981)	Basic dimerisation domain (EML4, 31–140)	415/89	[27].
		Tyr kinase (ALK, 555–822)		
**EML4-ALK (496; 1057)**	ALK_HUMAN (1620)	HELP/WD domains (EML4, 223–298, 299–327)	228/12	[51]
		Tyr kinase (ALK, 555–822))		
**TPR-MET (218; 1009)**	NP_003283.2 (2363)	Leu-zipper (TPR, 75–99, 117–141)	147/26	[25]
	MET_HUMAN (1390)	Tyr-kinase (MET, 287–546)		
**TFG-ALK (240; 1057)**	NP_006061 (400)	Coiled-coil dimerisation domain (TFG, 93–124)	175/138	[24]
	ALK_HUMAN (1620)	Tyr-kinase (ALK, 299–566)		
**TEL-JAK2 (337; 504)**	NP_001978.1 (452)	Sam dimerisation domain (TEL, 38–124)	558/203	[6].
	JAK2_HUMAN (1154)	Tyr-kinase (JAK2, 682–956)		
**EWS-ATF1 (264; 109)**	NP_005234.1 (656)	EAD (EWS, 1–86)	280/265	[28]
	NP_005162.1 (271)	Leu-zipper (ATF1, 366–425)		
**MLL-CBP (1362; 266)**	HRX_HUMAN (3969)	AT-hooks (MLL, 169–180, 217–227, 301–309)	2403/1620	[29]
	NP_004371.2 (2442)	HAT domain (CBP, 2412–2649)		
**MLL-ENL (1362; 92)**	HRX_HUMAN (3969)	AT-hooks (MLL, 169–180, 217–227, 301–309)	1436/1270	[7]
	NP_005925.2 (559)	Trans-activator helix (ENL, 1745–1829)		

We collected functional information for 9 fusion proteins, which suggests that disorder of the region intervening newly joined functional regions contributes to oncogenic function. The table shows the distance and length of disorder separating the two elements required for the oncogenic function.

(i) The first type is exemplified by BCR-ABL, where a Tyr-kinase phosphorylation motif in BCR gets fused with the Tyr-kinase domain within ABL [5]. Permanent phosphorylation of Tyr177 by ABL Tyr-kinase domain creates a binding site for the adapter protein Grb2 and sends continuous proliferation signals to the nucleus [5]. The phosphorylation site and kinase domain are 465 residues apart in the sequence, with a continuous stretch of disorder of about 257 amino acids (Figure 5A), which permits the chimera to fold back and undergo autocatalytic modification.

(ii) In the second type of mechanism, dimerisation and/or cytoplasmic relocalisation bring about permanent activation of receptor Tyr-kinases. In TFG-ALK, the TRK-fused gene (TFG) segment carries a coiled-coil dimerisation motif at a distance of 175 amino acids from the Tyr-kinase domain of ALK, which undergoes auto-activation due to phosphorylation and presents a novel binding site to SH2 domain containing signaling proteins [24]. The intervening region has 138 of its amino acids disordered, which is probably critical for enabling the Tyr-kinase domains to engage in multiple mutual intermolecular phosphorylation reactions (Figure 5B). A similar principle may apply to TPR-MET [25], TEL-JAK2 [6] and NPM-ALK [26]. In each case, the N-terminal part of the chimera provides the dimerisation domain, the C-terminal part contributes the Tyr-kinase domain, and two Tyr-kinases thus brought into vicinity will phosphorylate each other permanently.

EML4-ALK represents an interesting variation on this theme [27], because it is a special fusion protein that occurs in non-small-cell lung cancer, i.e. a solid tumor, where disorder appears to play a double role in pathological activation of the protein [27]. On the one hand, the basic domain of EML4 brings about the dimerisation of the fusion product, enabling the mutual phosphorylation of the Tyr-kinase domain of ALK and its phosphorylation region. On the other hand, EML4 also contains two other domains that are important for oncogenic activation, HELP and WD, which activate the Tyr-kinase domain via direct interaction.

(iii) In the third type of mechanism the fusion of a DNA-binding element to a trans-activator domain results in an aberrant transcription factor, where disorder enables the interplay of remote binding elements. For example, the fusion of Ewing sarcoma (EWS) oncogene with transcription factors ATF1 and Fli1 [10] creates oncogenic EWS fusion proteins (EFPs), which are potent transcriptional activators that combine the highly repetitive, disordered EWS activation domain (EAD) and the DNA-binding region of the fusion partner (Figure 5C). Their trans-activation function is located within the N-terminal 86 amino acids of EWS [28]. Thus, EFPs promote abnormal cellular growth due to deregulation of transcription of target genes. A similar mechanism of transformation may apply in the case of mixed-lineage leukemia (MLL) fusion products in hematological malignancies. MLL is involved in translocations with about 40 different partners [9], such as CREB-binding protein (CBP), a transcription co-activator. CBP has histone-acetyltransferase (HAT) activity, which is probably mis-targeted by the fused DNA-binding domains of MLL, three AT-hook motifs and a DNA-methyltransferase (DNMT) homology region. Critical for transforming activity in CBP are its HAT domain and the adjacent bromodomain [29], which cooperate in histone remodeling under the aberrant control of the DNA-binding region. When MLL gets fused with the transcription factor ENL [7] in myeloid leukemias, both the AT hooks and DNMT homology domain within MLL and the C-terminal transactivator domain of ENL contribute to the transactivator function of the fusion product.

## Discussion

Chromosomal translocations generating novel oncogenic fusion proteins represent the leading cause of neoplastic transformations in leukemias, lymphomas and sarcomas [1]–[4], but the structural characterization of proteins involved in fusion and/or the ensuing fusion proteins is largely incomplete thus far. Often, long regions of the proteins involved in the fusion event lack recognizable similarity to any other known protein, which is an indication of their likely structural disorder, as suggested in the case of CBP [8] and EFP [10]. Here we report that chromosomal translocations are highly correlated with structural disorder, and disorder also contributes to the oncogenic function elicited by the fusion event. Whereas translocating proteins tend to be longer than average human proteins, and longer proteins tend to be more disordered [19],[20], the elevated disorder of translocation proteins cannot be accounted for by their increased length. Rather, there is strong and specific selection for proteins with elevated structural disorder following translocation/fusion, which has many interesting implications.

The signs of selection at the protein level have already been suggested in a previous study on the non-random position of breakpoints in translocation genes [30]. The major findings, i.e. that the breakpoint is almost invariably located in-frame and there is only a limited and highly biased set of domain combinations in translocating proteins, point to selection forces that act at the level of proteins. Clearly, a protein that can be translated into a viable product which has functional advantages in cellular proliferation and clonal expansion, is significantly more likely to be observed in cancer. Our results on structural disorder of the protein chimera extend these findings and provide a structural rationale at the whole-protein level.

Our key conclusion is that structural disorder makes the fusion product of two unrelated proteins look like a natural protein that does not activate cellular degradation pathways. Joining two truncated globular domains at random would generate a folding-incompetent protein, unable to pass the quality control of the cell; thus it would be rapidly degraded without any chance to confer a proliferative advantage on the cell that harbors it. Because IDPs are depleted of hydrophobic amino acids [11],[12], the fusion of two IDRs does not expose more hydrophobic regions and does not cause major structural disturbances. Our findings are in accord with the suggestion that the cost the cell has to pay for the functional advantages conferred by structural disorder is that it is structurally permissive to joining unrelated proteins [8]. This is also underlined by the observation that breakpoints tend to avoid domains, the domains involved have an increased level of disorder and/or a close-to-normal ratio of hydrophobic residues exposed upon fusion. Overall, these preferences suggest that a high level and proper spacing of disordered regions increase solubility of truncated folding-incompetent segments probably in accord with an intramolecular chaperone effect [31].

In addition to survival in the cell, disorder may also be involved in the novel function(s) gained by fusion proteins. In several cases, the conformational freedom provided by long disordered segments enable the pathological interplay of remote functional elements brought together by the fusion event. There are several considerations in support of the role of disorder in these functions. For example, the transforming function of fusion products is rather insensitive to the actual position of the breakpoint, as shown by both the frequent occurrence of multiple breakpoints within the same protein, and a range of mutation studies where deletion of large regions of the fusion proteins leave their transforming activity intact [9],[10]. These observations are highly reminiscent of the insensitivity of the function of disordered proteins to deleting/scrambling their residues, which have led to the notion of fuzziness [32]. In addition, disorder is critical in the physiological functions of proteins analogous to the oncogenic functions discussed here. Autophosphorylation of remote regulatory elements enabled by disorder is a recurring theme in the activation of signaling Tyr-kinases, such as Src [33]. Dimerisation and mutual phosphorylation are reminiscent of the molecular mechanism of the activation of receptor Tyr-kinases [34]. Physiological transcription factors are also noted for the involvement and high level of disorder [14].

The elevated level of disorder in translocation proteins also suggests novel strategies of intervention against the ensuing cancers. Because IDPs often carry out their function by molecular recognition mediated by short recognition motifs, the interfaces of their binding partners resemble the active sites of enzymes or binding sites of receptors, thus they can be targeted by small-molecule inhibitors [35]. In addition, the segment around the site is structurally adaptable, which provides hopes for applying the Antibody-antigen Interaction Dependent Apoptosis (Aida) technique [36]. This technique relies on binding two specific caspase3-fused antibodies to a cancer-specific target, so that caspase can dimerise and auto-activate, eliciting an apoptotic response in the cell.

In conclusion, our work offers novel structural insight into the cellular survival and proliferative functioning of oncogenic fusion proteins generated by chromosomal translocations. If two proteins are joined within their IDRs, the chimera generated is likely to evade structural surveillance mechanisms of the cell and live long enough to manifest its altered function(s). This novel insight also raises some hope that interference with the emerging oncogenic function may be devised by taking the unique structural status of fusion proteins into consideration.

## Methods

### Acquiring and/or reconstructing the fusion proteins

Human chromosomal translocations and the relevant genes/proteins were collected by sifting through Swissprot, NCBI's GenBank and TICdb [37]–[39], searching the annotations for key expressions such as “chromosomal translocation”, “chromosome translocation” or “fusion protein”. Breakpoints and gene names were recorded when this information was available in the databank entries. We focused on protein-coding entries: whenever there was a partial peptide sequence in the annotation part of a GenBank fusion entry, we used NCBI's Tblastn [40] to compare the nucleotide sequence of the GenBank entry to the non-redundant set of all human proteins. The two most closely matching proteins (with percentage identity >95%), matching either end of the GenBank entry, were picked. This procedure resulted in 739 highly redundant protein identifiers. After comparing these proteins to one another with Blastp and replacing 42 gene names with their synonyms, the 739 proteins were found to belong to 305 different genes. We culled 101 more genes from the latest version of TicDB [39], altogether resulting in 406 translocation-related genes (Table S1, Supplementary material).

We next recorded the breakpoints in the annotation of the proteins or assigned them whenever translocation proteins from chimeric nucleic acid sequences in GenBank could be reconstructed; at least one breakpoint within the coding region could be identified in 146 genes. Using the transcript information in TicDB and the corresponding proteins in the Ensembl [41] database, the final number of translocation partner proteins increased to 255. Fusion proteins were next reconstructed from the chimeric nucleic acid sequences in GenBank by running Blastx to query NCBI's non-redundant protein database, and also from the coordinates provided by TicDB, which resulted in the reconstruction of 187 fusion proteins. These correspond to 171 non-redundant fusion sequences at a sequence identity threshold of 90%.

As controls, the sequences of experimentally verified IDPs were downloaded from the DisProt database [42] (http://www.disprot.org/) and fully ordered proteins were obtained from the PDB (http://www.rcsb.org/). A set of all human protein-coding genes and their transcript variants were obtained from the Ensembl website (http://www.ensembl.org). As of March 5, 2008, there were 22,297 protein-coding genes in the human dataset. We used as reference proteins the longest transcript for each human protein-coding gene. A set of human proteins (altogether 15,945) were also obtained from the Swissprot databank (http://expasy.org/sprot/) [38]. The Pfam domain database [43] was downloaded from the Pfam website (http://pfam.sanger.ac.uk/).

### Analysis of disorder and Pfam domains occurrence in the fusion proteins

Intrinsic disorder was predicted by the IUPred algorithm [17], which can predict disorder with a sensitivity of 76% at 5% false positive rate. Average percentage disorder was defined as the percent of amino acids with a disorder score ≥0.5.

Fusion proteins were analyzed for the occurrence of Pfam domains running Blastp against the entire set of Pfam-A domain sequences [43] and also the human subset of Pfam domains derived from Swissprot proteins. We set the thresholds for a domain match at an e-value<1e-06 and sequence similarity >60%. We found that at this similarity level there was less than 1% difference between the two sets of domain matches, so analyzed all 18,609 human Swissprot proteins for the occurrence of Pfam domains using only the Swissprot-derived human subset of Pfam. (Ideally, when looking for non-overlapping domain matches, choosing the best ones we would find only identity matches. We found that out of 29,848 non-overlapping Pfam domains in 14,541 human Swissprot proteins with at least one domain match there were only 1275 domain matches with less than 100% sequence similarity.) We further analyzed the translocation proteins with in-house Perl scripts, namely, (i) statistical significance of the difference between any two distributions was evaluated with the chi-square test; (ii) p-values corresponding to the calculated chi-square values and degrees of freedom were calculated by a computer program courtesy of Zsuzsa Dosztanyi; (iii) percentage values of disorder, length, etc. distributions were also calculated by own Perl scripts.

### Calculation of exposed hydrophobic surface in the truncated Pfam domains

Actual values for the accessible nonpolar surface area (Anp) of truncated domains were determined as follows: The C-terminus of the protein structure was gradually truncated and the actual values of Anp for the truncated fragments were determined with the CHASA program [22] as suggested by [44]. The theoretical values were calculated using the formula by Chothia [45] and Janin [46].

The truncated domain structures were drawn and annotated using NCBI's Cn3D program [47]. The high-resolution images in Figures 4 and S3 were created using the Polyview-3D server [48].

## Supporting Information

Figure S1 Translocation frequency as a function of gene length.
(1.45 MB TIF)

Figure S2 Translocation frequency as a function of protein disorder. Ratio of the number of all human proteins and translocation proteins with breakpoint as a function of disorder (%IU). The best fitting trendline is a parable.
(0.13 MB EPS)

Figure S3 Effect of truncation on the Interleukin 2 domain. (A) On the top the theoretical and actual values for the accessible nonpolar surface area (Anp) are shown, calculated as described for Figure 4; (B) Structure of the IL2 domain (pdb code: 1irl). The C-terminal portion missing due to the translocation is colored grey.
(1.29 MB TIF)

Table S1 List of proteins observed in chromosomal translocations. 151 proteins without (Table S1A) and 255 proteins with (Table S1B) breakpoint (latter also indicated). The percentage disorder is also shown.
(0.07 MB XLS)

Table S2 List of all truncated Pfam domains and their mapping to the fusion proteins using Blastp.
(0.01 MB TXT)
