# Supplementary figures and images for: Intrinsic Structural Disorder Confers Cellular Viability on Oncogenic Fusion Proteins
Source: PLoS Comput Biol. 2009 Oct 30;5(10):e1000552. doi: 10.1371/journal.pcbi.1000552 (PMC2768585; doi:10.1371/journal.pcbi.1000552)

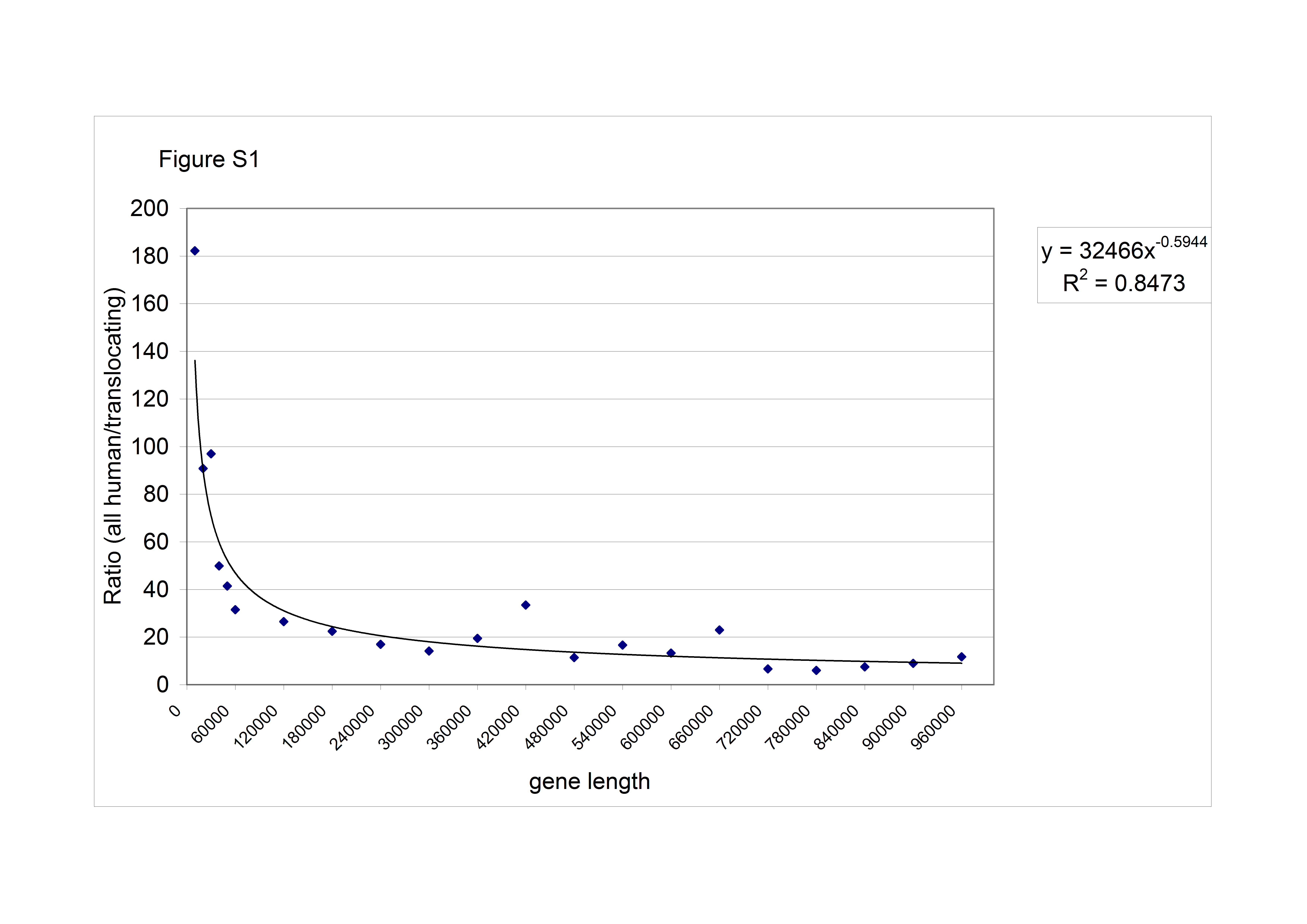

Supplement: Figure S1 — Translocation frequency as a function of gene length. (1.45 MB TIF) [file pcbi.1000552.s001.tif]
